# Structural basis of the heterodimerization of the MST and RASSF SARAH domains in the Hippo signalling pathway

**DOI:** 10.1107/S139900471400947X

**Published:** 2014-06-29

**Authors:** Eunha Hwang, Hae-Kap Cheong, Ameeq Ul Mushtaq, Hye-Yeon Kim, Kwon Joo Yeo, Eunhee Kim, Woo Cheol Lee, Kwang Yeon Hwang, Chaejoon Cheong, Young Ho Jeon

**Affiliations:** a Division of Magnetic Resonance Research, Korea Basic Science Institute, Ochang-eup Yeongudangiro 162, Cheongwon-gun, Chungbuk 363-883, Republic of Korea; b Division of Biotechnology, College of Life Sciences and Biotechnology, Korea University, Seoul 136-701, Republic of Korea; c College of Pharmacy, Korea University, Sejong-ro, Sejong 339-700, Republic of Korea

**Keywords:** Hippo signalling pathway, SARAH domains, MST, RASSF

## Abstract

Despite recent progress in research on the Hippo signalling pathway, the structural information available in this area is extremely limited. Intriguingly, the homodimeric and heterodimeric interactions of mammalian sterile 20-like (MST) kinases through the so-called ‘SARAH’ (SAV/RASSF/HPO) domains play a critical role in cellular homeostasis, dictating the fate of the cell regarding cell proliferation or apoptosis. To understand the mechanism of the heterodimerization of SARAH domains, the three-dimensional structures of an MST1–RASSF5 SARAH heterodimer and an MST2 SARAH homodimer were determined by X-ray crystallography and were analysed together with that previously determined for the MST1 SARAH homodimer. While the structure of the MST2 homodimer resembled that of the MST1 homodimer, the MST1–RASSF5 heterodimer showed distinct structural features. Firstly, the six N-terminal residues (Asp432–Lys437), which correspond to the short N-terminal 3_10_-helix h1 kinked from the h2 helix in the MST1 homodimer, were disordered. Furthermore, the MST1 SARAH domain in the MST1–RASSF5 complex showed a longer helical structure (Ser438–Lys480) than that in the MST1 homodimer (Val441–Lys480). Moreover, extensive polar and nonpolar contacts in the MST1–RASSF5 SARAH domain were identified which strengthen the interactions in the heterodimer in comparison to the interactions in the homodimer. Denaturation experiments performed using urea also indicated that the MST–RASSF heterodimers are substantially more stable than the MST homodimers. These findings provide structural insights into the role of the MST1–RASSF5 SARAH domain in apoptosis signalling.

## Abstract 2

The heterodimeric structure of the MST1 and RASSF5 SARAH domains is presented. A comparison of homodimeric and heterodimeric interactions provides a structural basis for the preferential association of the SARAH heterodimer.

## 1. Introduction   

Recent research into cellular homeostasis has shown that the Hippo signalling pathway, which is mediated by mammalian sterile 20-like kinase (MST, the human orthologue of Hippo), controls organ size in animals and regulates cell proliferation and cell death (Polesello *et al.*, 2006 ▶; Ikeda *et al.*, 2009 ▶; Del Re *et al.*, 2010 ▶; Avruch *et al.*, 2011 ▶). Mutation of Hippo kinase or Salvador (SAV) reveals a loss of function in growth restriction and apoptosis (Harvey *et al.*, 2003 ▶). The key players in this pathway include MST, Ras-association domain family (RASSF) proteins and WW45 (the human orthologue of Salvador), which are tumour suppressors and play significant roles in the immune system and in cardiovascular function (Saucedo & Edgar, 2007 ▶; Harvey & Tapon, 2007 ▶; Ling *et al.*, 2008 ▶).

**Figure 1:**
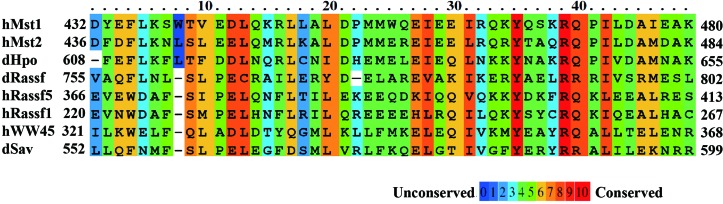
Sequence comparison of the SARAH domains of hMST1, hMST2, hWW45, hRASSF5, hRASSF1, dHPO, dSAV and dRASSF. Colours represent the degree of homology. Red blocks denote regions of sequence identity across all homologues. Partially conserved residues are shown in orange to blue accordingly. For all domain subfamilies, members from human (h) and *Drosophila* (d) are shown.

The MST-mediated apoptosis pathway is controlled by its interaction with RASSF family proteins and the WW45 protein (Feig & Buchsbaum, 2002 ▶; Khokhlatchev *et al.*, 2002 ▶; Praskova *et al.*, 2004 ▶; Callus *et al.*, 2006 ▶). Interestingly, all three classes of tumour suppressors, MST, RASSF and WW45, interact through their SARAH (SAV/RASSF/HPO) domains (Scheel & Hofmann, 2003 ▶; Fig. 1 ▶). SAV and HPO are from *Drosophila* and have the mammalian homologues WW45 and MST, respectively. RASSF is of human origin and its *Drosophila* orthologue is CG4656 or dRASSF (Polesello *et al.*, 2006 ▶). Moreover, colocalization of the interaction partners has been observed and has been shown to be dependent on these SARAH domains (Oh *et al.*, 2006 ▶; Ling *et al.*, 2008 ▶).

MST1, also called KRS-2 (Taylor *et al.*, 1996 ▶), is a serine/threonine kinase with 487 amino-acid residues (Creasy & Chernoff, 1995*b* ▶). Its catalytic domain is homologous to yeast STE-20 and belongs to the group II germinal centre kinase subgroup of the STE-20 kinase family (Creasy & Chernoff, 1995*b* ▶). MST1 is expressed ubiquitously and the protein is present in all of the human cell lines examined to date (Creasy & Chernoff, 1995*b* ▶). MST2 or KRS-1 (Taylor *et al.*, 1996 ▶) has 491 amino-acid residues and the sequence of its catalytic domain is 95% similar to that of MST1 (Creasy & Chernoff, 1995*a* ▶). Unlike MST1, MST2 shows high levels of expression in adult kidney, skeletal and placental tissues and very low expression in adult heart, lung, liver and brain tissues (Creasy & Chernoff, 1995*a* ▶). Hippo, the MST orthologue of *Drosophila*, is also a STE-20 family protein kinase and binds to and phosphorylates the tumour suppressor protein SAV, which is known to interact with the Warts (WTS) protein kinase (Wu *et al.*, 2003 ▶). Their interaction promotes WTS phosphorylation (Wu *et al.*, 2003 ▶).

The Hippo pathway is well defined in *Drosophila*; it is known to be involved in cellular homeostasis and controls cell proliferation and apoptosis (Halder & Johnson, 2011 ▶). Mutations in the genes involved in this pathway lead to tissue overgrowth and most of them have been identified as tumour suppressors (Harvey *et al.*, 2003 ▶). The pathway consists of a kinase cascade regulated by cell–cell contact and cell polarity that inhibits the transcriptional coactivator Yorkie (Avruch *et al.*, 2012 ▶). The core pathway components are the germinal centre (GC) kinase Hippo, which phosphorylates MOB as tumour suppressor (MATS)/MOB1 and large tumour suppressor (LATS) with the assistance of the scaffold protein SAV (Avruch *et al.*, 2012 ▶). Phosphor-LATS, after binding to phosphor-MATS, autoactivates and phosphorylates Yorkie, resulting in its nuclear exit (Avruch *et al.*, 2012 ▶). In mammals, the Hippo orthologues MST1 and MST2 utilize the Salvador orthologue WW45 to regulate the kinases LATS1/LATS2 and NDR1/NDR2 (NDR, nuclear dbf2-related kinase; Avruch *et al.*, 2012 ▶).

The RASSF family proteins RASSF1A and RASSF5 (NORE1) are known to be biochemical inhibitors of MST1 kinase. However, they can also activate MST1 kinase when they recruit MST1 into the membrane fraction and induce apoptosis through their interaction (Praskova *et al.*, 2004 ▶; Khokhlatchev *et al.*, 2002 ▶). Recently, it has been reported that the interaction between RASSF5 and MST1 mediates apoptosis in response to the death receptor ligands tumour necrosis factor (TNF) and TNF-related apoptosis-inducing ligand (Park *et al.*, 2010 ▶). Furthermore, RASSF proteins bind to Hippo in competition with SAV and thus inhibit SAV-mediated Hippo signalling (Polesello *et al.*, 2006 ▶).

Therefore, it has been suggested that the interaction between RASSF and MST family proteins has a crucial function not only in the activation of apoptosis but also in the silencing of death signalling (Saucedo & Edgar, 2007 ▶). The structure of the RASSF and MST binding interface is key to understanding the control mechanism in this apoptosis pathway.

We have previously solved the homodimeric structure of the MST1 SARAH domain by nuclear magnetic resonance (NMR) spectroscopy (Hwang *et al.*, 2007 ▶). We have also investigated the interactions of the SARAH domains from MST1, RASSF5 (NORE1) and WW45 by NMR perturbation experiments (Hwang *et al.*, 2007 ▶). Although the MST1 SARAH domain shows a helical structure that forms a homodimer in an antiparallel manner, the sharp kink of the N-terminal 3_10_-helix enables the kinase domains of each protomer to come together in close proximity for autophos­phorylation. A recent report on the murine RASSF5 SARAH domain structure showed a homodimeric interface similar to that of the MST1 SARAH domain but without the short N-terminal helix of the SARAH domain (Makbul *et al.*, 2013 ▶). Intriguingly, the homodimeric structure of the MST1 SARAH domain is disrupted when the RASSF5 SARAH domain is added to the complex. Instead, a stable heterodimeric structure is formed between the RASSF5 and MST1 SARAH domains with 1:1 stoichiometry. Thus, it is of great interest to understand how and why this homodimeric structure changes to a heterodimer and what the structural differences between the homodimer and heterodimer are.

In this study, we determined the heterodimeric structure formed by the MST1 and RASSF5 (NORE1) SARAH domains using X-ray crystallography. We also elucidated the MST2 homodimeric structure and performed structural and biophysical comparisons of the homodimeric and heterodimeric interactions. This information may provide detailed understanding of the protein–protein interaction through SARAH domains and structural insights into the mechanism underlying the regulation of Hippo signalling.

## 2. Materials and methods   

### 2.1. Protein expression and purification   

The human MST1 SARAH domain (residues 432–480) and the human RASSF5 SARAH domain (residues 366–418) were expressed in *Escherichia coli* strain BL21 as glutathione *S*-transferase (GST) fusion proteins and purified as described previously (Hwang *et al.*, 2007 ▶). Selenomethionine (SeMet)-labelled human MST1 SARAH domain was expressed in methionine-auxotroph B834 (DE3) cells (Novagen) that had been grown at 25°C overnight. For preparation of the MST1–RASSF5 SARAH heterodimer sample, the purified MST1 SARAH domain and RASSF5 SARAH domain were mixed in a 1:2 molar ratio and the MST1–RASSF5 SARAH heterodimer was separated from the mixture by gel-filtration chromatography (Superdex 75) equilibrated with 25 m*M* 4-(2-hydroxyethyl)-1-piperazineethanesulfonic acid (HEPES) pH 7.0, 100 m*M* NaCl, 2 m*M* dithiothreitol (DTT). Fractions containing the MST1–RASSF5 SARAH heterodimer were collected and concentrated to 20 mg ml^−1^. The selenomethionyl protein used for the single-wavelength anomalous diffraction (SAD) experiments was also prepared as described above. The human MST2 SARAH domain (residues 436–484) was expressed from *E. coli* strain BL21 as a GST fusion protein and was purified as described previously (Hwang *et al.*, 2010 ▶). The human MST2 SARAH homodimer was concentrated to 40 mg ml^−1^ in a crystallization buffer consisting of 25 m*M* HEPES pH 7.0, 100 m*M* NaCl, 2 m*M* DTT.

### 2.2. Crystallization and structure determination   

Native crystals of the human MST1–RASSF5 SARAH heterodimer were grown at 20°C using the hanging-drop vapour-diffusion method. Crystals were obtained by mixing the protein solution (20 mg ml^−1^) with the same volume of well buffer consisting of 35%(*v*/*v*) 2-methyl-2,4-pentanediol (MPD), acetate pH 4.8. The crystals belonged to space group *C*222_1_ and contained one heterodimer complex in the asymmetric unit. SeMet-labelled crystals of the human MST1–RASSF5 SARAH heterodimer were obtained from a reservoir solution consisting of 2 *M* ammonium sulfate, 3-(cyclohexylamino)-1-propanesulfonic acid (CAPS) pH 10.5, 0.2 *M* lithium sulfate. The crystals were cryoprotected using ethylene glycol at a final concentration of 20%(*v*/*v*) and were flash-cooled in a nitrogen stream at 100 K. The native and SAD data sets were collected using synchrotron radiation on beamline 4A at Pohang Accelerator Laboratory, Pohang, Republic of Korea and on beamline 17A at Photon Factory, Tsukuba, Japan. The structure of the human MST1–RASSF5 SARAH heterodimer was determined by the SAD method using the data collected from SeMet-labelled protein crystals and *SOLVE* (Terwilliger & Berendzen, 1999 ▶). The phase was further improved with *RESOLVE* (Terwilliger, 2000 ▶) and the initial model was built automatically by *ARP*/*wARP* (Langer *et al.*, 2008 ▶). All data sets were integrated and scaled using *HKL*-2000 (Otwinowski & Minor, 1997 ▶); the data-collection statistics are summarized in Table 1 ▶. The model was completed using iterative cycles of model building with *Coot* (Emsley & Cowtan, 2004 ▶) and refinement with *PHENIX* (Adams *et al.*, 2010 ▶). All final models were validated by *PROCHECK* (Laskowski *et al.*, 1993 ▶).

MST2 SARAH homodimer crystals were obtained using the hanging-drop vapour-diffusion method at 20°C with a protein concentration of 40 mg ml^−1^ and a reservoir solution consisting of 2.0 *M* NaCl, 8% polyethylene glycol (PEG) 6000. The crystals belonged to space group *P*4_1_2_1_2 and contained one homodimer complex in the asymmetric unit. The crystals were cryoprotected using ethylene glycol at a final concentration of 20%(*v*/*v*) and were flash-cooled in a nitrogen stream at 100 K. The native data set was collected using synchrotron radiation on beamline 17A at Photon Factory. All data sets were integrated and scaled using *HKL*-2000 (Otwinowski & Minor, 1997 ▶); the data-collection statistics are summarized in Table 1 ▶. The crystal structure of the MST2 SARAH homodimer was determined by the molecular-replacement method using the structure of the MST1–RASSF5 SARAH heterodimer as a search model with *Phaser* (McCoy *et al.*, 2007 ▶). The model was completed using iterative cycles of model building with *Coot* (Emsley & Cowtan, 2004 ▶) and refinement with *PHENIX* (Adams *et al.*, 2010 ▶).

### 2.3. Urea-induced denaturation   

Protein samples (0.2 mg ml^−1^) were equilibrated with 0–7.5 *M* urea in 10 m*M* sodium phosphate buffer pH 7.4 at 25°C. Protein unfolding was monitored by measuring the molar ellipticity at 222 nm at 298 K using a 1 mm path-length cuvette with a Jasco J710 spectropolarimeter at Korea Basic Science Institute. The data were fitted by nonlinear regression to a two-state model to obtain the free energy of unfolding (Santoro & Bolen, 1988 ▶).

### 2.4. Computational analysis   

Solvent-accessible surface area (ASA) was calculated for the MST2 homodimer and the MST1–RASSF5 heterodimer, and for individual monomers of MST1, MST2 and RASSF5, using *NACCESS* v.2.1.1 (http://www.bioinf.manchester.ac.uk/naccess), which is based on the method of Lee & Richards (1971 ▶). We used the default probe radius of 1.4 Å for the ASA calculations. H atoms were added to the MST1, MST2 and MST1–RASSF5 dimer structures and were subjected to 10 000 steps of conjugate-gradient energy minimization in vacuum dielectric using the CHARMM22_PROT force field and the CHARMM22 charge set in *VEGA ZZ* v.2.4.0 (http://nova.colombo58.unimi.it). The difference between the nonpolar ASA of the dimer and its monomers gives the nonpolar surface area buried at the interface (hydrophobic burial).

The energies of MST1, MST2 and MST1–RASSF5 were calculated from energy-minimized structures in *SYBYL-X* v.2.0 (http://tripos.com). The structures were subjected to energy minimization implementing the Gasteiger–Hückel charge set in dielectric 80 using the Powell algorithm and the Tripos force field for 10 000 iterations to a termination gradient of 0.05 kcal mol^−1^ Å^−1^.

Computational alanine scanning was performed on MST1 and MST2 homodimers and the MST1–RASSF5 heterodimer using a protocol described by Kortemme and coworkers (Kortemme & Baker, 2002 ▶; Kortemme *et al.*, 2004 ▶). This method identifies potential hotspots at protein–protein interfaces. We used a cutoff of >1.0 kcal mol^−1^ to identify the most important residues that contribute to binding and stability of the interface.

## 3. Results and discussion   

### 3.1. Structure of the MST1–RASSF5 SARAH heterodimer   

**Figure 2:**
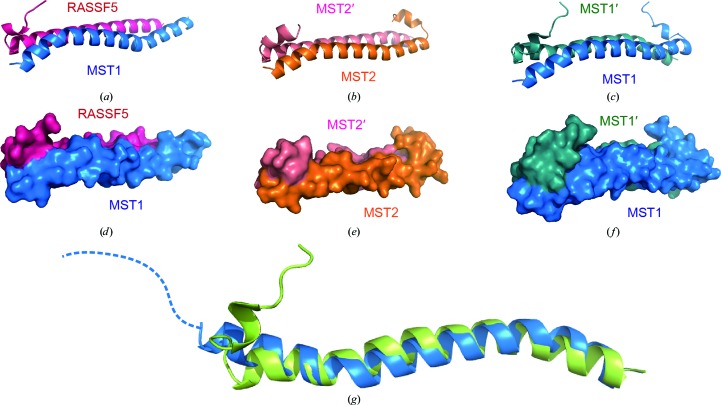
Overall structures of the SARAH dimers. Structures of the MST1–RASSF5 SARAH heterodimer (*a*, *d*), the MST2 SARAH homodimer (*b*, *e*) and the MST1 SARAH homodimer (*c*, *f*) are shown as ribbon diagrams and surface representations, respectively. For ease of comparison, the orientations shown in all panels are identical. For the MST1–RASSF5 SARAH heterodimer (*a*, *d*), the blue ribbon represents the backbone structure of the MST1 SARAH domain and the pink ribbon represents that of the RASSF5 SARAH domain. (*g*) Comparison of the monomer structures of the MST1 SARAH domain in the homodimer (light green) and in the heterodimer (blue). Note that the short h1 helix of the MST1 SARAH domain is missing and the length of the MST1 h2 helix is extended in the MST1–RASSF5 heterodimer.

The heterodimeric MST1–RASSF5 SARAH complex was prepared by mixing an excess amount of RASSF5 with the MST1 SARAH domain solution, followed by size-exclusion chromatography to separate the heterodimer fractions. As shown in Supplementary Fig. S1^1^, the excess RASSF5 eluted earlier than the heterodimer. We collected the fractions from the heterodimer peak for crystallization (fractions 40–42). We confirmed the 1:1 protein ratio of the MST1 and RASSF5 SARAH domains in the collected fractions by SDS–PAGE (Supplementary Fig. S1*b*). The crystal structure of the SARAH complex, consisting of the MST1 (residues 432–480) and RASSF5 (residues 366–418) SARAH domains, was determined by SAD from a single SeMet-derivatized crystal at the Se *K* edge. A single dimeric complex was found in the asymmetric unit in space group *C*222_1_. Clear electron density allowed residues Ser438–Lys480 of MST1 and residues Val365–Glu412 of RASSF5 to be traced, showing a tight heterodimeric complex (Figs. 2 ▶ *a* and 2 ▶ *d*). While the SARAH domain of RASSF5 consists of two helices (h1, residues Glu66–Ser373; h2, residues Ile374–Glu412), that of MST1 has only one helix which corresponds to the h2 helix in the MST1 homodimer structure (Hwang *et al.*, 2007 ▶). The electron density for the six N-terminal residues of the MST1 SARAH domain (Asp432–Lys437), which correspond to the short N-terminal 3_10_-helix h1 kinked from the h2 helix in the MST1 homodimer investigated in our previous study (Hwang *et al.*, 2007 ▶), was missing in the MST1–RASSF5 heterodimer (Fig. 2 ▶ *a* and Supplementary Fig. S2*b*). The helical structure corresponding to the h2 helix of the MST1 SARAH domain starts from Ser438 in the heterodimer, while that of the MST1 homodimer starts from Val441 (Hwang *et al.*, 2007 ▶). Thus, the h2 helix in the MST1–RASSF5 heterodimer is three residues longer than that in the MST1 homodimer. These observations suggest that the h1 helix of the MST1 SARAH domain becomes unstructured and that the h2 helix extends to the N-terminus when it binds to the RASSF5 SARAH domain; this was confirmed by NMR spectroscopy in solution in the current study. The backbone connectivity of residues Asp432–Ser438 is missing in the triple-resonance spectra owing to line broadening caused by chemical exchange or fast relaxation of the signals in the region (denoted by red arrows in Supplementary Fig. S2*a*). The unfolding of the MST1 SARAH h1 helix in the MST1–RASSF5 heterodimeric structure may result from steric hindrance of the MST1 h1 helix by the straight RASSF5 h2 helix, which is evident in superimposed structures of the homodimers and heterodimers (Supplementary Fig. S3).

Another difference in the MST1 structure between the heterodimer and the homodimer was that there was a significant distortion of the α-helix of the MST1 protomer in the MST1–RASSF5 heterodimer at proline residues Pro453 and Pro472. The angles of the flanking helical axes of Pro453 and Pro472 were 28.5 and 39°, respectively, which were much larger than those of the MST1 (9° and 32°, respectively) or MST2 (16° and 27°, respectively) homodimers (Supplementary Fig. S4). These sharp distortions provide larger binding interfaces than straight helices in the MST1 or MST2 homodimer. The main α-helix h2 of the RASSF5 protomer in the MST1–RASSF5 heterodimer is straight without proline residues at the corresponding positions (Supplementary Fig. S4*b*).

**Figure 3:**
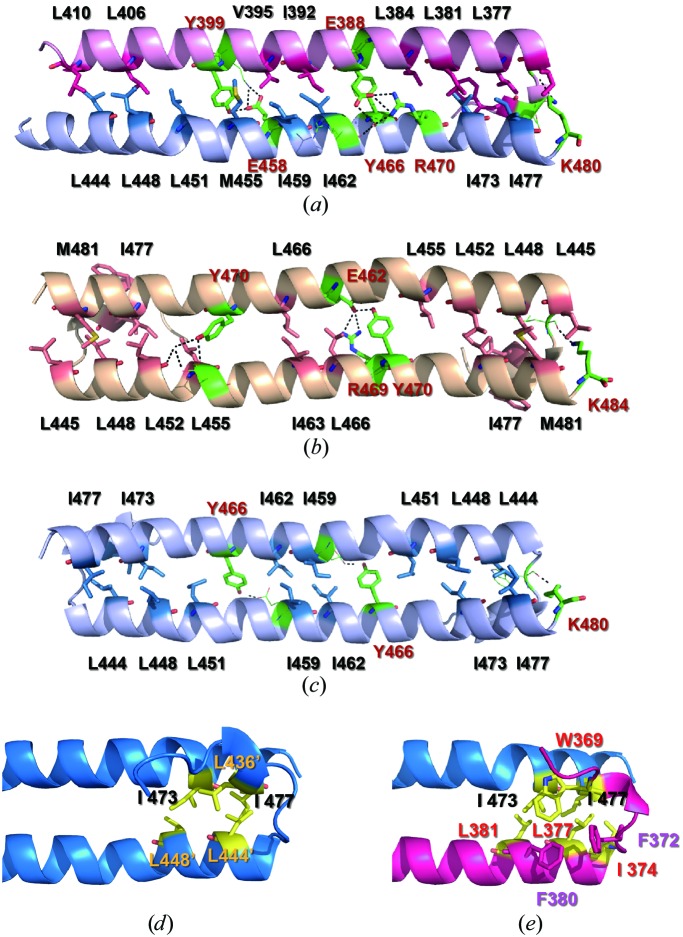
Comparison of the dimeric interactions of SARAH domains based on computational alanine scanning. (*a*, *b*, *c*) Ribbon representations depicting the side chains of residues having dimeric interactions derived from the computational alanine scanning of SARAH dimeric interfaces are shown for the MST1–RASSF5 SARAH heterodimer (*a*), the MST2 SARAH homodimer (*b*) and the MST1 SARAH homodimer (*c*). Residues with ΔΔ*G*_bind_ > 1.0 kcal mol^−1^ in computational alanine scanning are represented as stick models. Among the residues, Trp369, Ile374 and Glu387 of RASSF5 and Phe437 and Leu440 of MST2 are not seen in the figure and are not labelled for clarity. Residues that have polar interactions in the dimeric interface are shown in green. Red balls represent the water molecules mediating the hydrogen bonds between the two protomers. For the MST1–RASSF5 SARAH heterodimer (*a*), the light blue ribbon represents the backbone structure of the MST1 SARAH domain and the light pink ribbon represents that of the RASSF5 SARAH domain. (*d*, *e*) Ribbon representations with the side chains of the hydrophobic core formed by the N-terminal helix–turn–helix region are shown for the MST1 SARAH homodimer (*d*) and the MST1–RASFF5 SARAH heterodimer (*e*). Residues with ΔΔ*G*_bind_ > 1.0 kcal mol^−1^ in computational alanine scanning are shown as yellow sticks. Red sticks represent the side chains that interact with the aromatic residues in the hydrophobic core. Line models represent residues involved in the inter-protomer hydrogen bonding with smaller values of ΔΔ*G*_bind_ than 1.0 kcal mol^−1^.

The MST1–RASSF5 SARAH heterodimer is characterized by head-to-tail interaction of the two protomers, forming an antiparallel helix dimer between the elongated α-helix of MST1 SARAH and the h2 helix of RASSF5 SARAH. The dimer structure is stabilized by hydrophobic interactions and hydrogen bonds. 24 residues in the heterodimer exhibited ΔΔ*G* > 1.0 kcal mol^−1^ in computational alanine scanning (Fig. 3 ▶ *a* and Table 2 ▶): 12 residues from MST1, Leu444, Leu448, Leu451, Met455, Glu458, Ile459, Ile462, Tyr466, Arg470, Ile473, Ile477 and Lys480, and 12 residues from RASSF5, Trp369, Ile374, Leu377, Leu381, Leu384, Glu387, Glu388, Ile392, Val395, Tyr399, Leu406 and Leu410. On the other hand, only 18 residues in the MST1 homodimer (Hwang *et al.*, 2007 ▶) exhibited ΔΔ*G* > 1.0 kcal mol^−1^ in computational alanine scanning.

In addition, the side chain of Lys398 of RASSF5 was found to form a bifurcated hydrogen bond to Glu458 of MST1. Tyr399 of RASSF5 formed an additional hydrogen bond to Glu458. Moreover, the guanidine of Arg470 of MST1 formed a bifurcated hydrogen bond to the side-chain carbonyl of Glu388 of RASSF5. In turn, the carbonyl group of Glu388 formed a hydrogen bond to the backbone carbonyl of Arg463 of MST1 indirectly through a water molecule. Furthermore, the side-chain amino group of Lys480 of MST1 formed a bifurcated hydrogen bond to the backbone carbonyl groups of Asp370 and Phe372 of RASSF5 (Fig. 3 ▶ *a*). The total number of direct inter-protomer hydrogen bonds (including bifurcated hydrogen bonds) in the heterodimer was ten, whereas the numbers of hydrogen bonds in MST1 (Hwang *et al.*, 2007 ▶) and MST2 (see below) were three and eight, respectively (Supplementary Figs. S5*a*, S5*b* and S5*c*). Therefore, we think that the extensive hydrophobic and polar contacts in the MST1–RASSF5 complex stabilize the heterodimer structure better than they stabilize the homodimer structures.

The average distance of the two helical axes of the h2 helices in the MST1–RASSF5 heterodimer was found to be 10.0 Å, while those in the MST1 and MST2 homodimers were 10.2 and 10.8 Å, respectively. This result indicates that the two antiparallel helices in the MST1–RASSF5 heterodimer have tighter binding to each other than those in the homodimers. Comparison of the total energy of the h2 helices in the MST1–RASSF5 heterodimer and those in the MST1 and MST2 homodimers indicated a significant energy decrease when the homodimers changed to the MST1–RASSF5 heterodimer (Table 2 ▶). This is in accordance with the results of the denaturation experiments performed with urea (see below).

### 3.2. Structure of the MST2 SARAH homodimer   

The crystal structure of the MST2 SARAH homodimer (residues 436–484) is shown in Figs. 2 ▶(*b*) and 2 ▶(*e*). The structure was determined by molecular replacement starting from a MST1–RASSF5 SARAH heterodimer structure. The asymmetric unit was found to contain one dimeric complex in space group *P*4_1_2_1_2. The overall structure of the MST2 SARAH homodimer was very similar to that of the MST1 SARAH homodimer, although there were some minor differences in the local structures (Figs. 2 ▶ *b* and 2 ▶ *e*). Like the MST1 SARAH homodimer investigated in our previous study (Hwang *et al.*, 2007 ▶), the MST2 SARAH homodimer has an antiparallel helix dimer structure with a short N-terminal helix h1 folded toward h1′ of the dimeric binding partner. The main dimeric interactions consist of hydrophobic interactions of the nonpolar side chains and several inter-protomer hydrogen bonds. However, there are some characteristic differences between the MST2 SARAH domain and the MST1 SARAH domain.

Firstly, the structures of the two protomers were found to be slightly different from each other in the MST2 SARAH homodimer, while those in the MST1 SARAH homodimer were identical. The distortions of the main helix h2 at the conserved proline residues in the MST2 homodimer in protomer *A* were 16 and 27° at Pro457 and Pro476, respectively, while those in protomer *B* were 12 and 15° (Supplementary Figs. S4*d* and S4*e*). Thus, h2 of one protomer had a more linear structure than that of the other protomer. In addition to this difference, the hydrogen bonds between the h2 helices of the two protomers were not symmetric. Tyr470 in protomer *A* was found to be hydrogen-bonded to Glu462 in protomer *B*, and Tyr470 in protomer *B* was found to be hydrogen-bonded to Asp456 in protomer *A* (Fig. 3 ▶ *b* and Supplementary Fig. S6). When we overlaid the two protomers of the MST2 homodimer, we found that the side-chain conformations of Tyr470 and Arg474 in the two protomers differed significantly from each other (Supplementary Fig. S6*a*), resulting in the positioning of the hydroxyphenyl group at the other side of the dimeric interface, while most of the backbone and side-chain structures converged well, with a root-mean-square deviation (r.m.s.d.) of 0.81 Å for backbone atoms and 1.22 Å for heavy atoms. Moreover, the side chain of Arg474 in protomer *A* extended away from Tyr470 in the dimeric interface, but in protomer *B* it was in close proximity to Tyr470 (Supplementary Figs. S6*b* and S6*c*). The structural differences between the two protomers in the MST2 homodimer seem to arise from crystal-packing forces. There are several reported cases where the symmetry has been broken in the structures of homodimers; these have been reviewed by Brown (2006 ▶). It would be interesting to determine whether the symmetry of the MST2 SARAH homodimer also breaks in solution. The backbone amide signals in the heteronuclear single-quantum coherence (HSQC) spectrum of the MST2 SARAH domain have been assigned (Hwang *et al.*, 2010 ▶), but they comprised only one set of signals, which suggests that in solution the MST2 SARAH domain has one symmetric structure or is in a rapid dynamic equilibrium between two asymmetric structures. In addition, the short N-terminal helices h1 and h1′ in the MST2 homodimer have been identified as typical α-helices, while those in the MST1 homodimer are 3_10_-helices (Supplementary Figs. S4*c*–S4*f*).

Analysis of the well ordered water molecules in the dimeric interface showed that the MST1–RASSF5 SARAH heterodimer contains a larger number of water molecules mediating the inter-protomer hydrogen bonds than the MST2 SARAH homodimer (Supplementary Figs. S5*c* and S5*d*). Among the water molecules in the MST2 SARAH structure with *B* factor values below 25 Å^2^, only one water molecule mediates an inter-protomer interaction, while six water molecules in the MST1–RASSF5 SARAH structure were identified as mediators of inter-protomer hydrogen bonds. We think that the difference in the number of water-mediated polar contacts further explains the fact that the MST1–RASSF5 heterodimer has higher structural stability than the MST2 homodimer.

### 3.3. Computational alanine scan of SARAH dimeric interfaces   

To identify the ‘hotspots’ of the SARAH dimeric inter­actions, we performed computational alanine scanning (Kortemme *et al.*, 2004 ▶), which predicts which amino-acid side chains would destabilize the interface when mutated to alanine. Alanine scanning measures the effect of deletion of side-chain atoms beyond the C^β^ atom of an amino acid on the affinity of dimeric interaction. We used the protocol described by Kortemme *et al.* (2004 ▶). The critical residues that showed Gibbs free-energy changes of greater than 1.0 kcal mol^−1^ upon alanine substitution, *i.e.* ΔΔ*G*_binding_ > 1.0 kcal mol^−1^, are represented as stick models in Figs. 3 ▶(*a*), 3 ▶(*b*) and 3 ▶(*c*). Structural analysis showed that many hydrophobic and polar residues in the dimer interfaces affected the stability of the dimeric interaction. Among the conserved residues in the SARAH domains (Fig. 1 ▶), the mutation of tyrosine to alanine in the MST1–RASSF5 SARAH heterodimer produced the most dramatic effects in all of the dimer sets tested, with ΔΔ*G*_binding_ = 4.1 kcal mol^−1^. 17 nonpolar residues in the MST1–RASSF5 SARAH heterodimer had ΔΔ*G*_binding_ > 1.0 kcal mol^−1^, while 15 residues in the MST1 and 19 residues in the MST2 SARAH homodimers showed this change. For polar residues, seven amino acids in the MST1–RASSF5 heterodimer, three in the MST1 homodimer and five in the MST2 homodimer had ΔΔ*G*_binding_ > 1.0 kcal mol^−1^. This result reflects the fact that the number of residues that are energetically important for the stability of dimeric interaction in the MST1–RASSF5 SARAH heterodimer is substantially greater than that for the MST1 SARAH homodimer. In our previous study (Hwang *et al.*, 2007 ▶), protomers of the MST1 SARAH domain were found to spontaneously dissociate from their homodimeric binding partner to form a more stable heterodimer with the RASSF5 SARAH domain when MST1 was mixed with RASSF5. It is thought that the increased polar and nonpolar interactions in the MST1–RASSF5 SARAH domain are the main factors for the stabilization of the heterodimer.

A significant contribution to the heterodimerization of the SARAH domain is provided by the hydrophobic core formed by the N-terminal helix–turn–helix region of one protomer and the C-terminus of the h2 helix of the dimeric partner (Figs. 3 ▶ *d* and 3 ▶ *e*). We identified which of the critical residues forming the hydrophobic core had a ΔΔ*G*_binding_ larger than 1.0 kcal mol^−1^. In the MST1 homodimer, the hydrophobic core was found to be stabilized by the three leucine residues Leu436, Leu444 and Leu448 of one protomer and the two isoleucine residues Ile473 and Ile477 of the other protomer (Fig. 3 ▶ *d*). Two hydrophobic cores were symmetrically positioned at the two ends of the h2 helices in the MST1 homodimer. In contrast, the MST1–RASSF5 heterodimer had only one hydrophobic core, but had more extensive interactions stabilized by Trp369, Ile374, Leu377 and Leu381 of RASSF5 and Ile473 and Ile477 of MST1. In addition, the aromatic side chain of Trp369 was surrounded by the two phenylalanines Phe372 and Phe380, which further stabilized the hydrophobic core of the MST1–RASSF5 SARAH heterodimer (Fig. 3 ▶ *e*), while all of the residues in the hydrophobic core in the MST1 homodimer were aliphatic (Fig. 3 ▶ *d*).

### 3.4. Comparison of urea-induced denaturation curves of SARAH domains   

**Figure 4:**
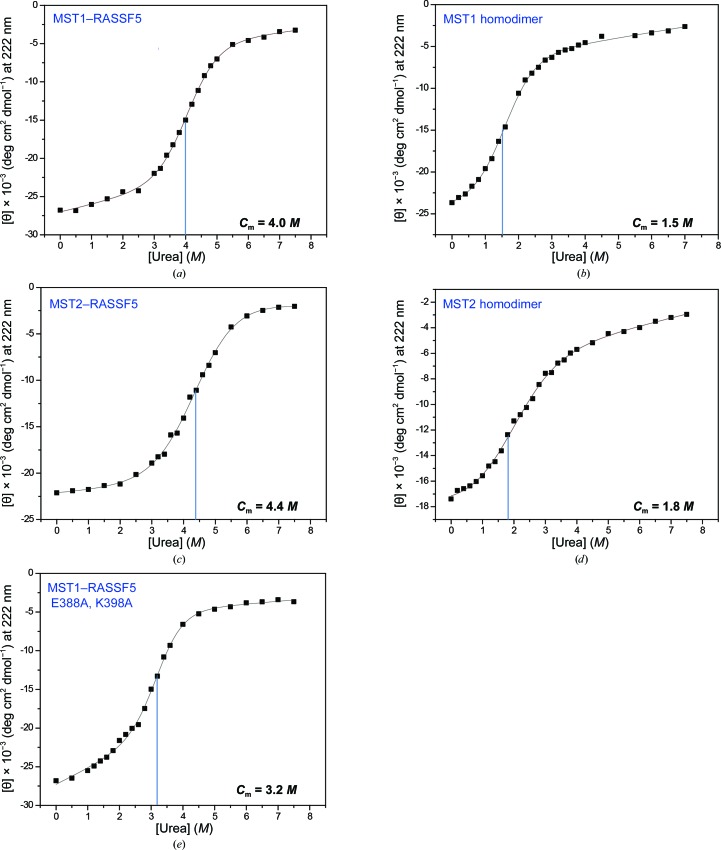
Comparison of the urea-induced denaturation curves of SARAH domains. The secondary-structural changes in the SARAH domains upon increases in the urea concentration were observed by monitoring the molar ellipticity [θ] at 222 nm in far-UV circular dichroism (CD) for the MST1–RASSF5 SARAH heterodimer (*a*), the MST1 SARAH homodimer (*b*), the MST2–RASSF5 SARAH heterodimer (*c*), the MST2 SARAH homodimer (*d*) and a double-mutant MST1–RASSF5 (E388A and K398A of RASSF5) SARAH heterodimer (*e*). Dotted lines indicate the transition concentration (*C*_m_) of urea for the denaturation of α-helical structures.

In our previous study (Hwang *et al.*, 2007 ▶), we analyzed NMR spectral changes and found that the two protomers of the MST1 SARAH homodimer readily dissociate to form heterodimers when they encounter the RASSF5 SARAH domain in solution. A similar result was obtained in a titration experiment of the MST2 SARAH homodimer with addition of the RASSF5 SARAH domain in solution (Supplementary Fig. S7). To compare the structural stabilities of the SARAH domains, we performed urea-induced denaturation while monitoring the far-UV circular dichroism (CD). By monitoring the molar residue ellipticity [θ] at 222 nm, we observed the secondary-structural changes that occurred upon urea-induced denaturation. Since SARAH domains consist of α-helices, the result indicates the transition concentration (*C*_m_) of urea for the denaturation of the α-helical structure. Fig. 4 ▶ shows the urea-induced denaturation curves of SARAH domains. We found that both the MST1–RASSF5 and MST2–RASSF5 heterodimers had higher *C*_m_ values than the MST1 and MST2 homodimers (Fig. 4 ▶). The results are in accordance with the structural observations.

We further analyzed the experimental results of urea-induced denaturation of SARAH domains and estimated the free-energy difference between homodimerization and heterodimerization by the linear extrapolation method for the denaturation experiment (Pace & Shaw, 2000 ▶; Lee *et al.*, 2010 ▶; Santoro & Bolen, 1988 ▶):
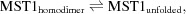

*C*_m_ = 1.5 *M* (urea concentration), Δ*G*° = 2.0 ± 0.23 kcal mol^−1^; 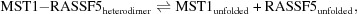

*C*_m_ = 4.0 *M* (urea concentration), Δ*G*° = 4.8 ± 0.33 kcal mol^−1^.

From the urea-induced denaturation profiles of MST1–RASSF5_heterodimer_ and MST1_homodimer_, it was found that MST1–RASSF5_heterodimer_ is more stable than MST1_homodimer_, with free energies of unfolding of 4.8 ± 0.33 and 2.0 ± 0.23 kcal mol^−1^, respectively. This result suggests that the heterodimeric interaction is much stronger than the homodimeric interaction.

When we introduced a double mutation (E388A and K398A) into RASSF5, the stability of the MST1–RASSF5 SARAH heterodimer was greatly reduced to a *C*_m_ of 3.2 *M* urea (Fig. 4 ▶ *c*). The effect of mutations in the urea denaturation experiment is in good agreement with the results of the analyses of the SARAH dimer interface by computational alanine scanning.

These results indicate that MST–RASSF heterodimers are more stable than MST homodimers and that heterodimer formation is preferred when MSTs encounter RASSFs in solution. Thus, it is reasonable that MSTs preferentially form heterodimers with RASSFs, resulting in the regulation of their activities and interactions with other binding partners such as SAV or RAF1.

## 4. Conclusion   

The RASSF family proteins, which are tumour suppressors, function as both biochemical inhibitors of MST kinases and activators when they recruit MST1 into the membrane fraction and induce apoptosis (Praskova *et al.*, 2004 ▶; Khokhlatchev *et al.*, 2002 ▶). Elucidation of the complex structures of RASSF and MST proteins is critical to understand the mechanism underlying the regulation of apoptosis and organ-size control.

By analyzing the three-dimensional structures of the SARAH domains of the MST1–RASSF5 heterodimer and the MST2 homodimer, together with the previously determined structure of the MST1 homodimer, we identified the key contributing affinities, including hydrophobic interactions and polar contacts. In addition, their relative contributions to the stability of the dimer were evaluated by computational alanine scanning. In the MST1–RASSF5 heterodimer, the extensive interactions by the ‘hotspot’ residues, including aromatic and polar side chains, provide a rationale for the stability of the heterodimer. Our results strongly support the previous observations that the full-length MST and RASSF proteins form a heterodimer under physiological conditions (Khokhlatchev *et al.*, 2002 ▶; Romano *et al.*, 2010 ▶). The preferential formation of the heterodimer may be a key mechanism in regulation in the Hippo signalling pathway.

**Figure 5:**
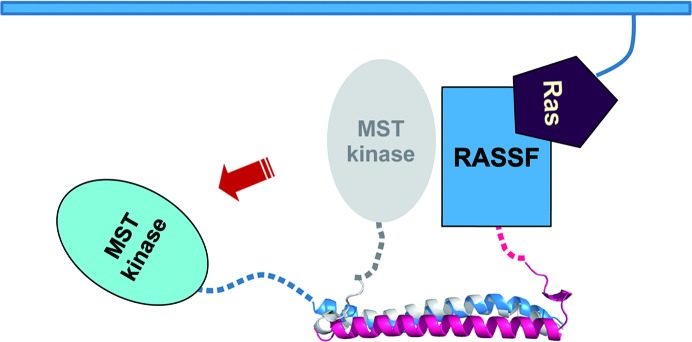
Schematic model showing how the MST1 kinase domain gains motional freedom by heterodimerization with RASSF5 in the membrane environment. The MST1 SARAH promoter undergoes structural change when it binds to the RASSF5 SARAH domain to form a heterodimer. By extension of the h2 helix and unfolding of the h1 helix, the MST1 SARAH domain in the heterodimer can provide motional freedom to the MST1 catalytic domain. The grey ribbon represents the MST1 SARAH domain in the homodimer and the blue ribbon denotes the MST1 SARAH domain in the heterodimer with RASSF5. The magenta ribbon represents the RASSF5 SARAH domain in the heterodimer with MST1. The blue horizontal bar represents a cellular membrane where the prenylated Ras protein anchors.

It is notable that the MST1 SARAH promoter undergoes structural change when it binds to the RASSF5 SARAH domain to form a heterodimer. By extension of the h2 helix and unfolding of the h1 helix, the MST1 SARAH domain in the heterodimer can provide motional freedom to the MST1 catalytic domain (Fig. 5 ▶). This motional freedom in turn may enable the MST1 catalytic domain to adopt a proper orientation for the phosphorylation of downstream effectors such as LATS1/LATS2 or NDR1/NDR2. Further studies on mutations in the MST1 SARAH domain that affect the motional freedom of the catalytic domain and its effects on the phosphorylation of the downstream effectors are required.

During the course of the preparation of our manuscript, the structure of the MST2–RASSF5 SARAH heterodimer was published (Ni *et al.*, 2013 ▶). The researchers performed systematic mutations of the residues in the MST2 SARAH domain to identify the critical residues for MST2 homodimerization and their effects on MST2–RASSF5 heterodimerization. Their results for some of the residues in the dimeric interface correspond to our findings, and their structures are complementary to our results based on the structures of the MST1 homodimer, the MST2 homodimer and the MST1–RASSF5 heterodimer. The main aspect of this study that is distinct from other previous studies is the comparison of the homodimeric and heterodimeric SARAH domain structures to provide structural insights into the change in the dimeric partner of the MST SARAH domain to the RASSF SARAH domain, which enables these structures to play their role in the apoptosis pathway.

## Supplementary Material

Supporting Information.. DOI: 10.1107/S139900471400947X/wa5070sup1.pdf

PDB reference: MST1–RASSF5 SARAH heterodimer, 4oh8

PDB reference: MST2 SARAH homodimer, 4oh9

## Figures and Tables

**Table 1:** Data-collection and structure-refinement statistics

	MST1RASSF5	MST1RASFF5 (SeMet)	MST2
Data collection			
Resolution ()	502.2 (2.282.20)	501.9 (2.001.90)	501.7 (1.761.70)
Space group	*C*222_1_	*I*4	*P*4_1_2_1_2
Wavelength ()	0.98000	0.97919	0.98000
Unit-cell parameters			
*a* ()	27.94	59.69	30.844
*b* ()	85.84	59.69	30.844
*c* ()	92.79	69.52	196.413
= = ()	90	90	90
Molecules per asymmetric unit	1	1	1
Multiplicity	4.0 (2.6)	4.3 (4.3)	10.1 (4.6)
*R*_merge_ (%)	7.9 (20.6)	7.3 (26.3)	5.3 (20.6)
Mean *I*/(*I*)	14.3 (6.0)	10.5 (3.9)	19.2 (6.0)
Completeness (%)	96.9 (85.8)	99.3 (98.9)	93.7 (68.2)
Refinement			
*R*_work_/*R*_free_	0.2167/0.2778		0.2346/0.2722
R.m.s.d., bond lengths ()	0.008		0.007
R.m.s.d., bond angles ()	1.122		1.09
Ramachandran plot			
Favoured regions (%)	98.80		98.90
Allowed regions (%)	1.20		1.1
Disallowed regions (%)	0		0
Average *B* factor (^2^)			
Chain *A*	19.61		21.45
Chain *B*	17.54		20.94
Water	19.28		23.89

**Table 2:** Analyses of dimer structures ASA, solvent-accessible surface area (^2^); *A*_np_, interface nonpolar area (^2^); *G*_binding_, free-energy changes for alanine mutation (kcalmol^1^); ND, not detected.

	MST1	MST1RASSF5	MST2
Calculation of interface area			
ASA of chain *A*	5367.2	4549.6	5174.5
ASA of chain *B*	5349.6	4846.8	5120.5
ASA of complex (chains *A* and *B*)	7858.5	7069.3	7316.0
Interface area of complex (chains *A* and *B*)	2858.3	2327.1	2979.0
Nonpolar surface area of chain *A*	3297.3	2922.0	3324.9
Nonpolar surface area of chain *B*	3336.3	2998.8	3308.6
Nonpolar surface area of complex (chains *A* and *B*)	4266.6	4051.4	4239.3
Interface nonpolar area (*A*_np_)	2367.0	1869.4	2394.2
Energy for h2 helices (kcalmol^1^)			
Van der Waals energy	589.142	628.296	622.624
Electrostatic energy	8.645	9.171	8.323
Total energy	158.142	221.464	200.955
Computational alanine scan			
No. of residues with *G*_binding_ > 1.0 kcalmol^1^	18	24	24
No. of polar residues with *G*_binding_ > 1.0 kcalmol^1^	3	7	5
Polar contacts			
No. of inter-protomer direct hydrogen bonds	3	10	8
No. of water molecules mediating the inter-protomer interaction	ND	6	1

## Footnotes

1 Supporting information has been deposited in the IUCr electronic archive (Reference: WA5070).
