# Supplementary material for: Structural basis of the heterodimerization of the MST and RASSF SARAH domains in the Hippo signalling pathway
Source: Acta Crystallogr D Biol Crystallogr. 2014 Jun 29;70(Pt 7):1944–53. doi: 10.1107/S139900471400947X (PMC4089488; doi:10.1107/S139900471400947X)
Supplement: Supplementary file 1 [file d-70-01944-sup1.pdf]

Acta Crystallographica Section D

Volume 70 (2014)

Supporting information for article:

Structural basis of the heterodimerization of the MST and RASSF SARAH domains in the Hippo signalling pathway

Eunha Hwang, Hae-Kap Cheong, Ameeq Ul Mushta, Hye-Yeon Kim, Kwon Joo Yeo, Eunhee Kim, Woo Cheol Lee, Kwang Yeon Hwang, Chaejoon Cheong and Young Ho Jeon

S1. Supplementary methods

S1.1. Nuclear magnetic resonance (NMR) spectroscopy

For uniform labeling with ^{15}N, or with ^{15}N and ^{13}C, cells were grown in M9 minimal medium supplemented with $^{15}NH_4Cl$ without or with ^{13}C-labeled glucose, respectively. NMR titrations were performed by recording ^{1}H-^{15}N NMR heteronuclear single quantum coherence (HSQC) spectra of ^{15}N-labeled MST2 SARAH upon addition of unlabeled RASSF5 SARAH. The ^{1}H-^{15}N HSQC spectra of all proteins were measured on a Bruker 800 MHz NMR spectrometer at the Korea Basic Science Institute (Ochang, Korea). The NMR measurements were performed with 0.3 mM ^{15}N-labeled protein with 25 mM 4-(2-hydroxyethyl)-1-piperazineethanesulfonic acid (HEPES), pH 7.0, 100 mM NaCl, and 2 mM dithiothreitol (DTT) containing 10% D_2O at 25 ℃. All NMR spectra were processed with Topspin 3.0 and analyzed with the SPARKY 3.1 program.

For preparation of the MST1-RASSF5 SARAH heterodimer NMR sample, the purified ^{13}C, ^{15}N-labeled MST1 SARAH domain and the RASSF5 SARAH domain were mixed in a 1:2 molar ratio, and the mixture was loaded onto a gel filtration column (Superdex 75) equilibrated with 25 mM HEPES, pH 7.0, 100 mM NaCl, and 2 mM DTT. Fractions containing the MST1-RASSF5 SARAH heterodimer were collected and concentrated to 1 mM. NMR measurements were performed at 35 °C on 1 mM ^{13}C,^{15}N-labeled MST1-RASSF5 SARAH heterodimer samples in 25 mM HEPES, pH 7.0, 100 mM NaCl, and 2 mM DTT containing 10% D_2O, using a Bruker 800 MHz NMR spectrometer. ^{1}H, ^{15}N, and ^{13}C resonance assignments were obtained from the following three-dimensional heteronuclear correlation experiments: CBCA(CO)NH, HNCACB, HN(CA)CO, HNCO, HN(CO)CA, HNCA, HBHA(CO)NH. All NMR spectra were processed with Topspin 3.0 and analyzed with SPARKY 3.1.

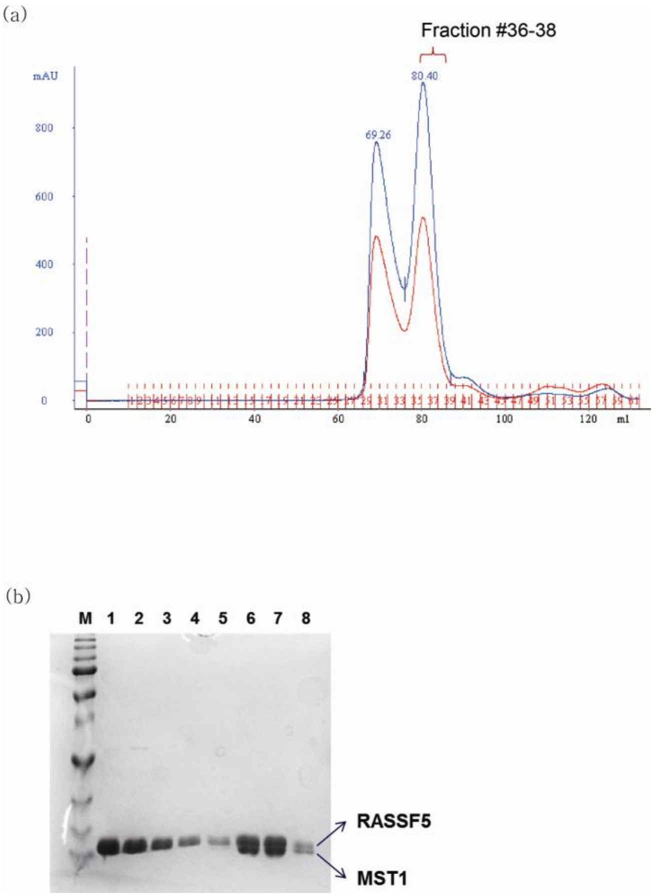

Figure S1 Size exclusion chromatography of the mixture of MST1 and RASSF5 SARAH domains with excess RASSF5 concentration (MST1 : RASSF5 = 1 : 2 molar ratio). (a) Size exclusion chromatogram. Peak at the retention volume 69.26 mL corresponds to RASSF5 homo-oligomer and peak at 80.40 mL corresponds to MST1-RASSF5 heterodimer. Fractions No. 36 to 38, collected for crystallization, were denoted on the peak. Superdex 75 column (GE Healthcare) was used. (b) SDS-PAGE analysis for the eluted fractions from size exclusion chromatography. M denotes marker, and lanes 1 to 4 were the fractions No. 30 to 33 and the lanes 5 to 8 correspond to the fractions No. 35 to 38, respectively.

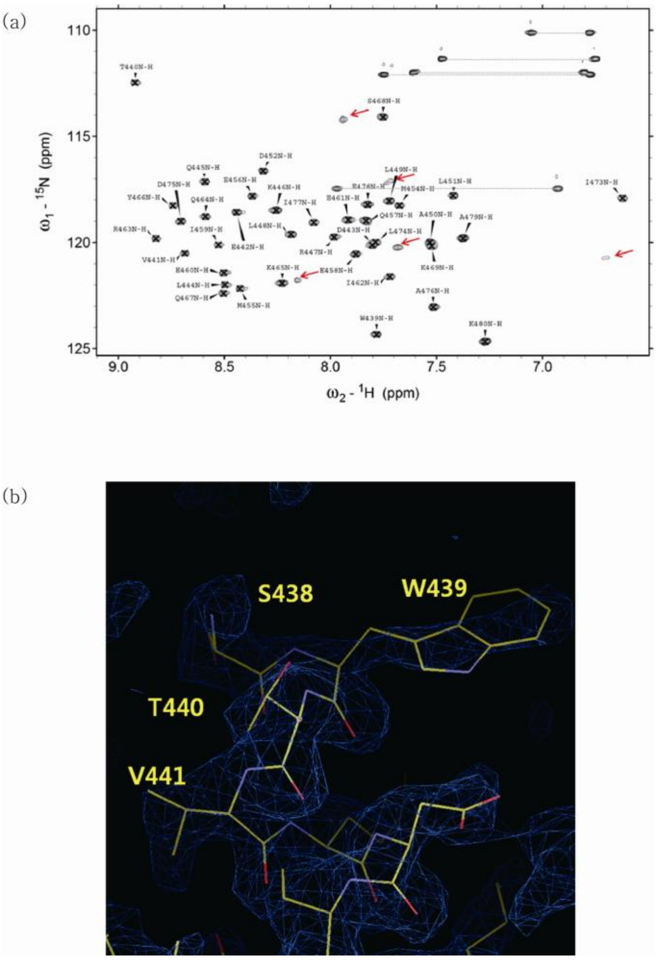

Figure S2 (a) Backbone assignment of the ^{13}C, ^{15}N-labeled MST1-RASSF5 heterodimer in the ^{1}H–^{15}N HSQC spectrum. ^{1}H–^{15}N side-chain pairs of Gln residues are connected by horizontal dotted lines. Red arrows indicate cross-peaks that could not be assigned because of missing sequential connectivity in the triple-resonance spectra. (b) Electron density $2F_o$–F_c map around the N-terminal missing region (residues D432–K437) of the MST1 protomer in the MST1-RASSF5 heterodimer at the 1.0 σ level.

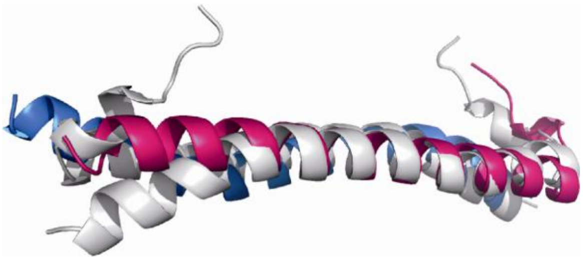

Figure S3 Superimposed structures of the MST1 SARAH homodimer and the MST1-RASSF5 SARAH heterodimer. Gray ribbons represent the MST1 SARAH domain in the homodimer, and the blue ribbon denotes MST1 SARAH domain in the heterodimer with RASSF5. The magenta ribbon represents the RASSF5 SARAH domain in the heterodimer with MST1.

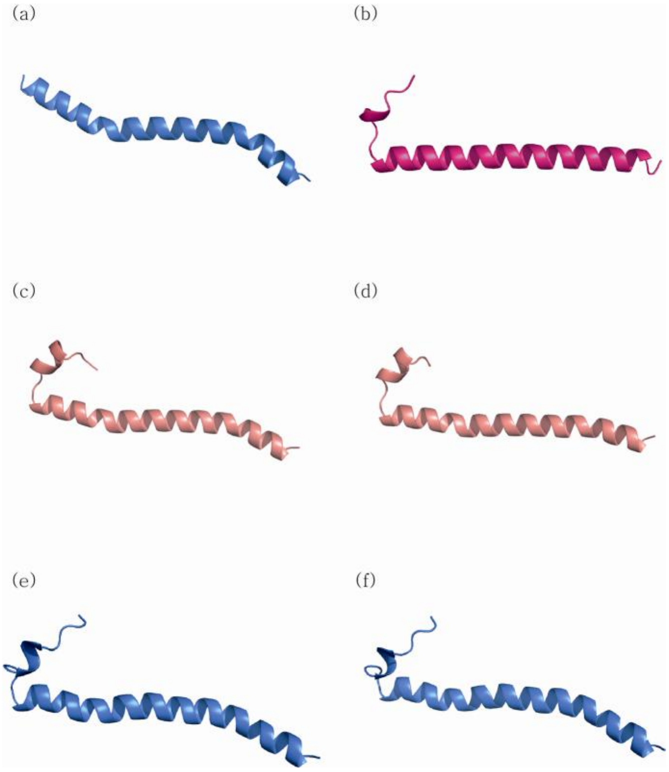

Figure S4 Comparison of the helical distortions in the SARAH domains. Ribbon representation of the structures of the MST1 protomer in the MST1-RASSF5 SARAH heterodimer (a), the RASSF5 protomer in the MST1-RASSF5 SARAH heterodimer (b), protomer A and protomer B in the MST2 SARAH homodimer (c and d), and the MST1 protomer in the MST1 SARAH homodimer (e).

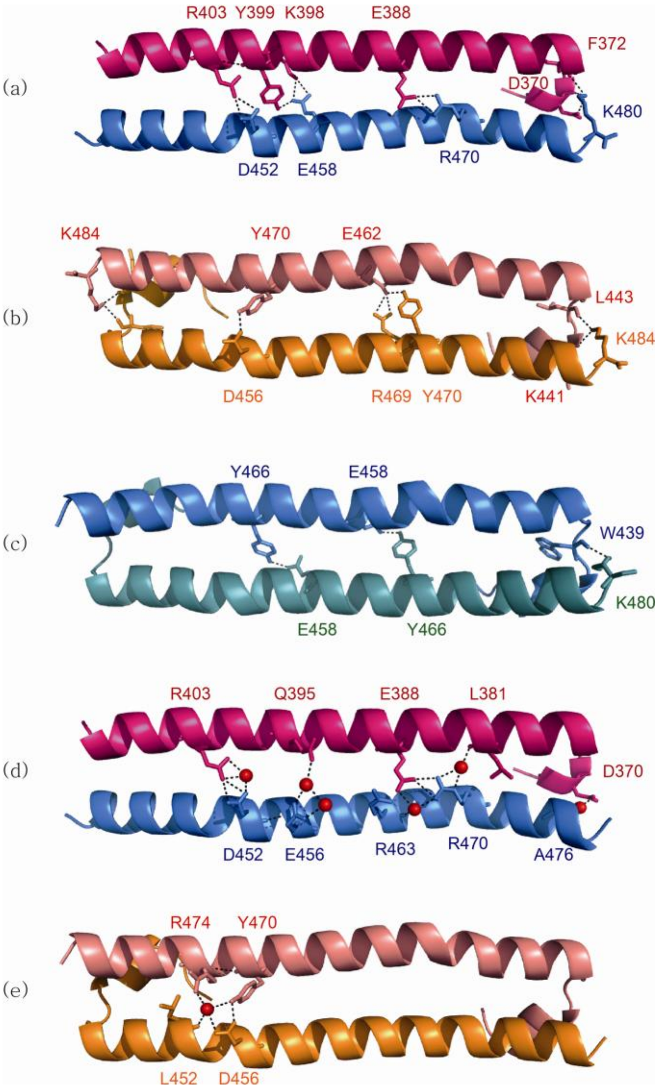

Figure S5 (a-c) Comparison of the direct interactions in the MST1-RASSF5 SARAH heterodimer (a), the MST2 SARAH homodimer (b), and MST1 SARAH homodimer (c). (d-e) Comparison of the bound waters mediating the polar contacts in the MST1-RASSF5 SARAH heterodimer (d) and the MST2 SARAH homodimer (e). Dots represent the inter-protomer hydrogen bonds. Amino acid numbers for the residues involved in the polar contacts are labeled.

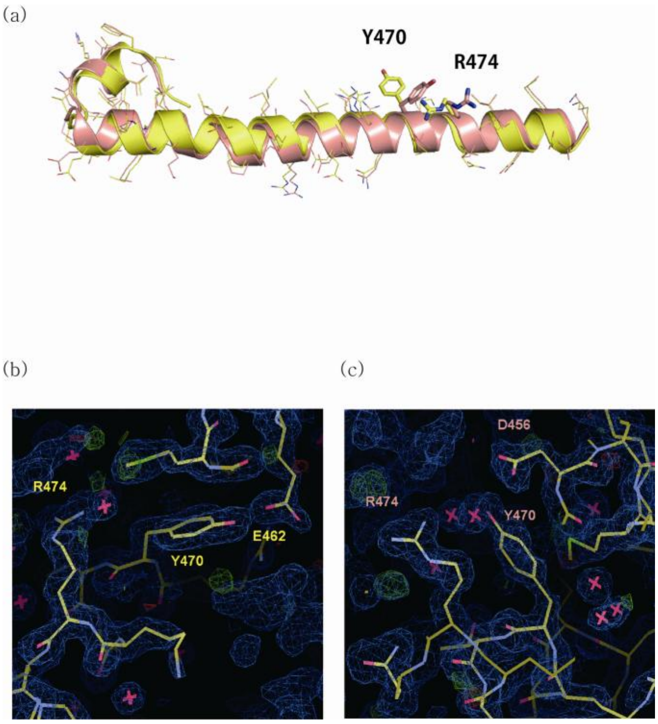

Figure S6 (a) Superposition of the two protomers in the MST2 SARAH homodimer. Protomer A and protomer B in the MST2 SARAH homodimer are indicated by yellow and pink, respectively. (b) Electron density $2F_o$–F_c map around the region (Y470 and R474) of the protomer A (left) and protomer B (right) in the MST2 SARAH homodimer at the 1.0 σ level.

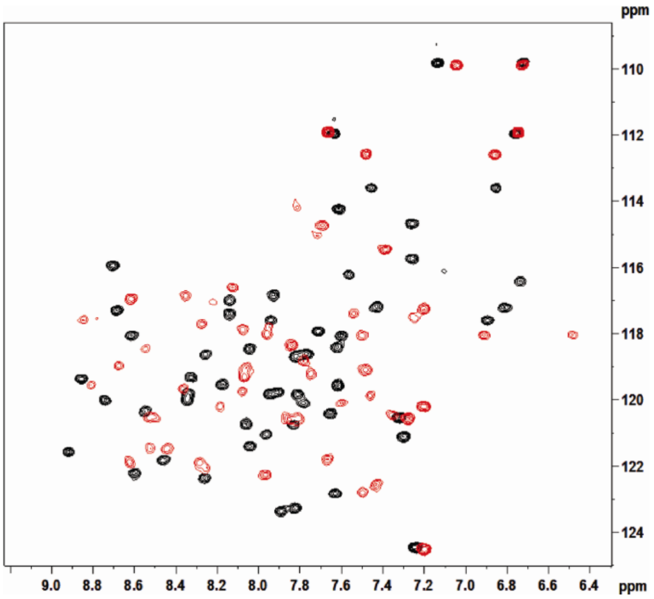

Figure S7 Binding of the human MST2 SARAH domain (436–484) to the human RASSF5 SARAH domain (366–418). Chemical shift perturbation after addition of 0.3 mM of the human RASSF5 SARAH domain to 0.3 mM of the ^{15}N-labeled human MST2 SARAH dimer. The chemical shifts are monitored in ^{1}H, ^{15}N correlation spectra. The spectrum of the free protein is shown in black, and the spectrum after addition of RASSF5 SARAH domain is shown in red.
